# Would it be possible to have better ways and routes of administration for vaccines against COVID-19?

**DOI:** 10.1590/1516-3180.2021.0316.18052021

**Published:** 2021-08-02

**Authors:** Álvaro Nagib Atallah, Samir Arbache

**Affiliations:** I MD, PhD. Full Professor and Head of the Discipline of Emergency Medicine and Evidence-Based Medicine, Universidade Federal de São Paulo (UNIFESP), São Paulo (SP); and Director of Cochrane Brazil, São Paulo (SP), Brazil; II MD. Preceptor of Dermatology, Universidade de Mogi das Cruzes (UMC), São Paulo (SP), Brazil; and Doctoral Student, Postgraduate Program on Evidence-Based Healthcare, Department of Medicine, Universidade Federal de São Paulo (UNIFESP), São Paulo (SP), Brazil

Dear Editor,

While celebrating with joy the evidence of the effectiveness of several vaccines against coronavirus disease 2019 (COVID-19), we are faced with the reality that billions of people in all regions of the planet will need to be vaccinated in order to achieve plausible control of the pandemic.

Scientific debates on which vaccine would be the best for COVID-19 have been given priority by scientific publications and news reports, with less emphasis than necessary on discussion of what the best vaccine administration routes might be.

Until such time that better evidence is available, we believe that research on new routes of administration is as important as proving the individual effectiveness of each vaccine. In these times of a pandemic, this is an opportunity to carry out randomized clinical trials to test different types of intradermal immunization in comparison with intramuscular immunization.

As a covering organ, the skin has mechanisms to protect the organism against physical, chemical or biological aggressions. Keratinocytes, Langerhans cells, fibroblasts, T lymphocytes, macrophages and dendritic cells are the first line of defense in the immune, cellular or humoral response.^1^

This immunological defense is not observed in the hypodermis or in the underlying muscles. Consequently, it is assumed that cutaneous microbiomes would have the function of “educating” the body’s immune system.^2^

Over recent years, injection of vaccines into the intradermal compartment has been considered. The logic is that vaccines injected into the dermis would provide greater immune stimulation.^2^

Several techniques for percutaneous immunizations have been described, among which three can be listed: superficial manual punctures with microneedles; intradermal Mantoux injection (the Mantoux method, which was first described in 1910 and has become the clinical standard for intradermal injection); and “DNA tattooing”, described by Bins et al.in 2005.^3^ In our view, this last method could be the most effective technique in a national vaccination campaign.^3^

What are the differences between these intradermal vaccination techniques?

Manual punctures with microneedles consist of dripping the vaccine on the skin and puncturing the site with needles. The diffusion of the vaccine in the dermis is passive.^2^^,^^4^^,^^5^ Assuming that two dozen perforations should be performed on the skin of each patient, the procedure could be exhausting for the technician, thus losing effectiveness.^6^

Intradermal injection with a syringe and needle (Mantoux method) requires technical skill. Moreover, the volume that needs to be injected is greater, with the aim of forming a bolus.

DNA tattooing^3^ uses tattoo machines to inject vaccines into the skin instead of inks. This vaccine delivery method was inspired by the ancient technique of artistic dermopigmentation. It was innovated and implemented by Brazilian dermatologists eight years ago, under the name MMP (Portuguese-language acronym for Micro-Infusion of Drugs into the Skin).^4^^-^^5^

Tattoo machine procedures are accurate and reproducible. The protocol for injecting medications with tattoo machines has already been established, consisting of 570 perforations per cm^2^, with a needle speed of between 60 Hz and 120 Hz and a needle depth of 300 microns.^5^ The rapid movement of the needles causes diffusion and active dispersion of the vaccine in the dermis.^4^ This gives rise to a greater area of immunological exposure that may extend to regional lymph nodes.^7^

We estimate that to immunize a patient, we need to puncture 1 cm^2^ of skin. We performed an experimental assay (unpublished results) in which we injected 1.0 ml of saline into artificial skin using a tattoo machine. This amount was enough to cover 20 to 50 cm^2^ of artificial skin, meaning that 20 to 50 patients could be immunized with this volume, depending on the skill of the technician. 

We propose to launch randomized, well-designed clinical trials that will aim to describe the effectiveness of intradermal vaccination in its three forms and compare them with each other and with traditional intramuscular vaccination.

These studies may be carried out in different scenarios and be brought together later, in syntheses of appropriate evidence.

It is essential to carry out analyses on cost-effectiveness and budgetary impacts, and to explore the possibilities of attaining greater efficiency, in order to cope with limited resources and enable better access to treatments for less developed countries.

Through such studies, data for clinical decisions can be provided, so as to be better prepared to face the challenges of comprehensive worldwide immunization; and, of course, to improve patients’ experience.
